# “Study Natural” without Drugs: An Exploratory Study of Theory-Guided and Tailored Health Campaign Interventions to Prevent Nonmedical Use of Prescription Stimulants in College Students

**DOI:** 10.3390/ijerph17124421

**Published:** 2020-06-19

**Authors:** Zhaohui Su, Michael Mackert, Xiaoshan Li, Jiyoon (Karen) Han, Brittani Crook, Benjamin Wyeth

**Affiliations:** 1 School of Nursing, University of Texas Health Science Center at San Antonio, San Antonio, TX 78229, USA; 2 Center on Smart and Connected Health Technologies, University of Texas Health Science Center at San Antonio, San Antonio, TX 78229, USA; 3 Mays Cancer Center, University of Texas Health Science Center at San Antonio, San Antonio, TX 78229, USA; 4 Stan Richards School of Advertising & Public Relations, Moody College of Communication, The University of Texas at Austin, Austin, TX 78702, USA; mackert@utexas.edu; 5 Department of Population Health, Dell Medical School, The University of Texas at Austin, Austin, TX 78702, USA; 6 Center for Health Communication, Moody College of Communication, The University of Texas at Austin, Austin, TX 78702, USA; 7 Program of Public Relations and Advertising, Beijing Normal University-Hong Kong Baptist University United International College, Zhuhai 519087, China; xiaoshan.li@utexas.edu; 8 Department of Telecommunications, Ball State University, Muncie, IN 47306, USA; jhan@bsu.edu; 9 Center for Health Communication, The University of Texas at Austin, Austin, TX 78702, USA; brittani@utexas.edu; 10 Brand Creative, The Richards Group, Inc., Dallas, TX 75204, USA; benwyeth@gmail.com

**Keywords:** campaign interventions, nonmedical use of prescription stimulants, NMUPS, theory-guided, tailored, college students, social norms, persuasive strategies

## Abstract

Nonmedical use of prescription stimulants (NMUPS) among college students continues to rise. While some anti-NMUPS campaigns are available, little is known about the campaign development process and how well college students evaluate these messages. To bridge this gap, we developed theory-guided anti-NMUPS campaign interventions that are tailored to college students’ characteristics and evaluated students’ response towards them. A total of 445 college students (74.4% female; *Mean*_age_ of 20; 18 to 35 years old) reviewed the campaign interventions and offered their evaluation via an online survey. Findings indicate that students responded to the campaigns positively. Results also indicate that female students are more likely to perceive the campaigns as effective than their male counterparts. Overall, the findings of this study suggest that theory-guided and tailored anti-NMUPS campaigns have great potential in changing students’ attitudes and behavior towards NMUPS. While this study fills critical gaps in the literature, considering the progress needed to strengthen the research field, more research is needed to further identify effective strategies that could prevent college students’ participation in NMUPS activities.

## 1. Introduction

Nonmedical use of prescription stimulants (NMUPS) is prevalent among college students and has been well documented in the literature [1,2]. Evidence shows that approximately 10.8% of college students have used prescription stimulants such as Adderall, a drug that is often prescribed to treat attention deficit hyperactivity disorder (ADHD), for non-medical reasons [2,3]. Extensive research has observed the detrimental role of NMUPS in damaging college students’ academic performance [4] and more alarmingly, students’ short-term and long-term health [5,6,7]. Despite control and regulation, college students’ NMUPS activities have been on the rise [8,9,10].

In the context of this study, NMUPS could be understood as college students’ use of prescription drugs without a prescription or in ways that are not consistent with the doctor’s instructions (e.g., extremely high doses) [11]. To address college students’ rising engagement in NMUPS activities, many universities and college campuses have been working to address this issue, using methods such as on-campus health campaigns. For instance, Valdosta State University used the 2010 National Red Ribbon campaign with the theme “I am Drug Free. Lock Your Meds”, aiming to reduce, if not eliminate, students’ NMUPS activities [12]. However, while useful insights are available, little to no theoretical evidence can be gained from these interventions regarding (1) how these campaigns are designed and (2) how college students evaluate these campaigns. As limited empirical findings can be obtained from existing campaigns to guide future intervention design, research is needed to bridge this gap in the literature.

## 2. Background

### 2.1. The Importance of Theory-Guided Health Communication Campaigns

Health communication campaigns are effective in changing individuals’ health-related beliefs and behavior [13,14,15,16]. Findings from a meta-analysis of health campaign studies from 1966 to 2012 indicate that health campaigns can increase knowledge and encourage positive behavioral changes in the target audience [14]. To further improve health campaign outcomes, scholars suggested that campaign interventions should incorporate theory into health campaign design and development, as theoretical frameworks could add structure to research and have the potential to boost campaign outcomes [17,18,19,20,21]. Adopting theoretical frameworks in research design can also improve interventions’ comparability and replicability, which have the potential to pave the way for subsequent systematic review studies, and in turn, contribute to insights into what is missing in the current literature and what is needed to further the research field [22,23,24].

Theory can be understood as ‘‘a body of logistically interconnected propositions which provides an interpretative basis for understanding phenomena’’ [25]. Extensive research suggests that theory-guided health campaign interventions are effective in changing college students’ risky health practices, such as smoking [26], alcohol abuse [27], unsafe sexual behavior [28], and lack of physical activity [29]. While a consensus has been reached regarding the importance of theory in health campaign research [20,21], there is a lack of empirical studies on theory-guided health campaigns conducted in the context of NMUPS [30,31,32]. In other words, limited understanding is available on how college students evaluate theory-guided anti-NMUPS campaigns that are tailored to their characteristics and preferences. Considering that different health topics and campaign strategies often result in varying intervention outcomes [33,34,35], insights into college students’ evaluation of theory-guided and tailored campaign interventions conducted specifically in the NMUPS context are needed.

### 2.2. “Justified Use” of NMUPS in College Students

Research suggests that college students lack adequate and accurate knowledge regarding risks associated with NMUPS [4,36]. More alarmingly, evidence shows that college students often underestimate the risks and overestimate the potential “benefits” of adopting NMUPS [36,37,38]. One potential contributing factor to this worrying on-campus NMUPS climate is how prescription stimulants are perceived by college students. Rather than deemed as dangerous and detrimental, prescription stimulants are widely exalted as “study drugs,” “a study tool,” or “smart pills” among college students [39,40,41]. For instance, Teter and colleagues found that 59.8% of college students who participate in NMUPS have done so because they believe prescription stimulants could “help [them] study” [42]. In the same vein, Desantis and Hane concluded that college students hold a favorable attitude towards NMUPS, to the extent that they believe prescription stimulants are “not a drug,” “all good,” and are morally acceptable to be used as a “study tool” [43].

Findings from a meta-analysis further reveal that the main reasons for college students’ participation in NMUPS are (1) to improve sports or academia-related performance, (2) to control sleep cycle and reduce anxiety levels, and (3) to assist with managing current health conditions [44]. That is, overall, only a small portion of college students’ NMUPS activities are associated with recreational purposes (e.g., use in parties to get high) [4,44,45]. These findings are very alarming, as college students’ “justified use” of NMUPS may further fuel their participation in NMUPS and in turn, harm their long-term academic performance and wellbeing [46,47]. Thus, to address these issues, on-campus anti-NMUPS campaign interventions are needed to change students’ attitudes and behavior towards NMUPS. One way to alleviate persistent and prevalent on-campus health crises such as NMUPS, as research indicates, is via integrating tailored persuasive techniques, such as campaign strategies developed based on social norms and students’ characteristics, into campaign design and development [48,49].

### 2.3. Social Norms and NMUPS

One well-accepted function of health campaigns is their ability to induce positive attitudinal and behavioral changes in individuals by changing the overall social norms surrounding the health practice [45,50,51]. Social norms could be understood as “shared expectations of culturally appropriate and desirable behavior” [52]. Due to the close-knit structure of college environments, social norms have a pronounced influence on students’ participation in NMUPS [45,53,54]. In general, social norms impact college students’ attitudes and behavior towards NMUPS in two ways: (1) shaping the macro sociocultural environment in which college students are immersed in (e.g., the permissive college culture on NMUPS) [55,56,57] and (2) influencing the micro social networks which college students are closely surrounded by (e.g., friends and family’s enabling attitude towards NMUPS) [45,54,58,59]. In terms of macro-level influence, evidence suggests that on-campus social norms play an important role in shaping college students’ NMUPS-related attitudes and behavior [45,60,61]. Research finds that compared to their same-age peers, full-time college students are more likely to participate in NMUPS activities [60,62]. Findings also suggest that young adults are more likely to participate in NMUPS during their time in college than their away-from-college years [60].

On a micro level, social norms shared by intimate social ties also influence college students’ attitudes and behavior towards NMUPS [4,45,61]. Evidence shows that college students’ perceptions and behavior towards NMUPS are often associated with how their intimate social contacts perceive and interact with NMUPS [45]. Findings suggest that more than 60% of college students surveyed obtained their prescription stimulants from their friends and peers [63]. Accumulated insights suggest that intimate social ties’ influence on college students’ beliefs and behavior towards NMUPS is multifaceted: college students who participate in NMUPS often (1) believe their social ties approve their NMUPS behavior [46,54], (2) have social contacts who also engage in NMUPS behavior [64], and (3) obtain their prescription stimulants from these social connections [45,65,66]. Social norms shaped by the intangible college culture and tangible social ties could further explain why progress that aims to address college students’ participation in NMUPS is difficult to solidify. One way to better understand the role of social norms in shaping college students’ participation in NMUPS as well as their potential response to anti-NMUPS campaigns is structured understanding guided by frameworks such as the Health Belief Model (HBM) and the Theory of Planned Behavior (TPB).

### 2.4. The HBM and the TPB Theoretical Principles

The HBM framework is one of the most utilized theoretical models in the field of health communication, which offers critical insights that could help explain and predict health behavior in various research contexts [67,68,69,70]. In health promotion or disease prevention research, the HBM has been widely adopted in guiding studies that aim to increase people’s knowledge and change their health-related attitudes and behavior [71,72,73,74]. The underlying logic of the HBM is that people are conscious individuals who would partake in goal-oriented health behavior upon receiving message cues that are relevant to them [70,75,76]. That is, the HBM proposes that people will take health actions if (1) they believe an adverse outcome could be avoided or a positive one could be achieved, (2) they believe by taking a recommended action, their behavior could generate positive outcomes, and (3) they believe they can successfully carry out a recommended health action [68,69,70,75,77].

Similar to the HBM, the TPB framework is also a value-expectancy theory. In other words, TPB is also founded on the premise that individuals’ beliefs and attitudes guide their behavioral changes [78,79]. Specifically, the TPB posits that people’s (1) attitudes and perceptions about a behavior, (2) beliefs related to their significant others’ acceptance of the behavior, and (3) perceptions about their ability to carry out the behavior shape their behavioral intention and in turn, their actual adoption of the behavior [78,79]. Overall, the TPB framework has been widely adopted by researchers in the field of health communication, such as studies conducted in the context of NMUPS [46,47,80]. For instance, using the TPB model as the guiding framework, researchers found that college students’ attitudes and subjective norms are significant predictors of their intention to change their NMUPS behavior.

In sum, both the HBM and the TPB frameworks propose that (1) individuals’ beliefs and attitudes could lead to their behavioral changes and (2) social norms play an important role in shaping people’s attitudes and behavior towards health practices [70,77,78,79]. By adopting the HBM and the TPB frameworks in guiding the design and development of campaign interventions, the purpose of this study is to investigate the following research question:

- How would college students evaluate tailored anti-NMUPS campaign interventions developed under the theoretical guidance of the HBM and the TPB frameworks?

We believe important understandings could be gained by answering the research question raised above, as there is an urgent need to fill the existing research gap and identify approaches that could effectively address and alleviate students’ engagement in NMUPS activities [81,82,83]. In addition, there is also a need to develop theory-guided interventions that can be replicated and applied in other health contexts to further strengthen the campaign intervention literature [84,85,86,87,88,89].

## 3. Methods

### 3.1. Participants and Procedure

A total of 528 students participated in this study; 445 of them provided usable responses. To ensure research rigor, an estimate of the margin of error to reach a 95% confidence level was calculated by 1/√N, where N equals the number of participants in a study. In this study, a fraction of 1/√445 returned an estimated margin of error of 0.05 (i.e., only a 5% chance of the sample results will be different from the true population), which is a widely adopted and recommended margin estimate [90]. Participants were recruited via a large participant pool consisting of students studying at The University of Texas at Austin (74% female; Mean age = 20, 18 to 35 years old). The majority of the participants were sophomore or older, and 61% of the participants were white. Participants received course credit for completing the survey. Alternative extra credit options were also offered to the participants. Study approval was obtained from the University’s Institutional Review Board (IRB number: 2012-10-0164). Students were asked to give their consent prior to their participation in the study by clicking “Agree” to the IRB information presented in the survey (This study was granted an exemption from obtaining a written consent form). After viewing the anti-NMUPS advertising campaigns, respondents were asked to participate in a survey that gauged their behavioral beliefs, emotions, normative beliefs, perceived realism, and control beliefs related to NMUPS. This online survey took approximately 15–25 min to complete.

### 3.2. Campaign Development

**Figure 1:**
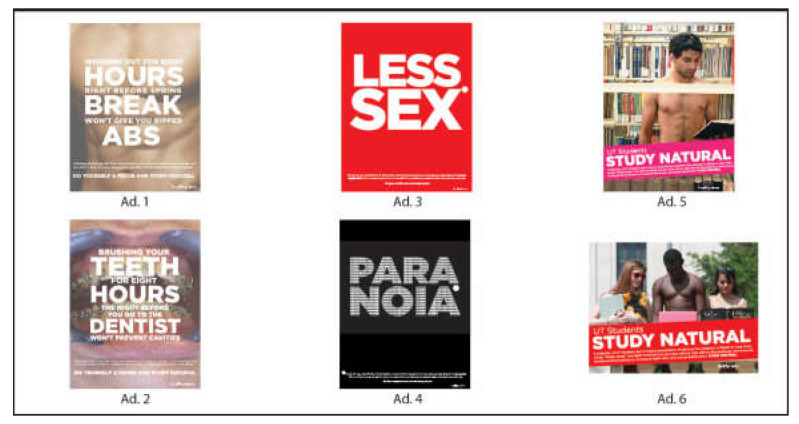
Health campaign interventions designed based on the Health Belief Model (Ad No. 1 to Ad No. 4) and the Theory of Planned Behavior (Ad. No. 5 and Ad. No. 6) frameworks. Ad: Advertisement.

In terms of creating tailored NMUPS campaigns, our study mainly focused on addressing the most problematic characteristics of college students’ NMUPS activities identified in the literature: (1) not understanding the long-term consequences of NMUPS participation, (2) lack of a clear picture of the severity of these prescription stimulants, and (3) being influenced by the negative social norms surrounding the practice of NMUPS [9,14,38,60,61,91]. The first two approaches, long-term consequences (i.e., the “Working Out” (Advertisement or Ad No. 1) and the “Brushing Your Teeth” ads (Ad No. 2) and severe negative outcomes (i.e., the “Less Sex” (Ad No. 3) and the “Paranoia” ads (Ad No. 4)) are primarily guided by the HBM framework [70,92]. For the third approach, which is guided by the TPB principles [78,79], we developed the “Study Natural-Library” (Ad No. 5) and the “Study Natural-Outdoors” ads (Ad No. 6) (see Figure 1).

The “Working Out” ad (Ad No. 1) and the “Brushing Your Teeth” ad (Ad No. 2) (see Figure 1) illustrate the flawed logic of choosing short-term benefits of abusing prescription stimulants over their long-term damages on personal health and quality of life. For example, the “Brushing Your Teeth” ad (Ad No. 2) relates NMUPS to a common logic flaw: just like brushing your teeth for eight hours before going to the dentist will not help prevent cavities, participate in NMUPS for short-term academic gains is unrealistic as well. To further heighten the persuasive powers of the ads, each ad’s headline was followed by an explanatory text: “And using Study Drugs (like Ritalin and Adderall) won’t make you smarter. In fact, long-term use can result in drug tolerance, changes in how you learn, and can negatively impact your grades,” and a succinct call to action: “Do yourself a favor and study natural.”

The “Less Sex” (Ad No. 3) and the “Paranoia” ads (Ad No. 4) (see Figure 1) aim to persuade students to realize NMUPS’s negative impacts on their health and quality of life. These ads focus on two common side-effects of the medications: sexual dysfunction and paranoia. Each of these ads contains a large simple, yet impactful headline (e.g., Less Sex) paired with an explanatory text to enhance the argument: “Study Drugs (like Ritalin & Adderall) are habit-forming and can have serious side effects like paranoia, not to mention sexual dysfunction, increased blood pressure, increased heart rate, and nervousness.”

Based on the TPB principles, we developed the “Studying Natural-Library” (Ad No. 5) and the “Studying Natural-Outdoors” (Ad No. 6) ads (see Figure 1), aiming to provide the audience with evidence on why they should avoid using prescription stimulants and persuasive cues that inform them of the ease of efforts it takes for them to not participate in NMUPS. These two ads focus on social norms by highlighting the fact most university students choose not to participate in NMUPS. Each message contains a tailored statement based on evidence on college students’ susceptibility to on-campus social norms, “A majority of UT (The University of Texas at Austin) students don’t misuse prescription medications like Adderall or Ritalin to help them study” to underscore the positive social norms that most college students adhere to regarding NMUPS, aiming to customize the campaign messages to challenge and change students’ established NMUPS perceptions. These ads then list key side effects associated with NMUPS to strengthen the argument, followed by a call to action to further increase interventions’ persuasive powers. It is worth noting that each of the six ads contains the logo of the university, which have the potential to elevate these interventions with added trustworthiness and acceptability [93,94].

### 3.3. Study Measures

#### 3.3.1. Demographic variables

Respondents were asked to provide information about their gender, age, ethnicity, and school classification (Table 1).

#### 3.3.2. Dependent Variables

Perceived effectiveness was measured using six items. Participants were asked to indicate their agreement with the statements by choosing one of the four available scales (1 = “definitely no” to 4 = “definitely yes”) adopted from Fishbein and Associates [95,96]. An example item was, “Was the message convincing?” (α = 0.93).

- Perceived realism was measured using two items (1 = “definitely no” to 4 = “definitely yes”): “Was the message believable?” and “was the message honest” (α = 0.76) [95,96].
- Negative emotional response was measured using four items (1 = “Not at all” to 4 = “very much”): “sad,” “angry,” “afraid,” and “disgusted” (α = 0.79) [95,96].
- Positive emotional response was measured using two items = “Not at all” to 4 = “very much”): “happy,” and “excited,” (α = 0.88) [95,96].

#### 3.3.3. Statistical Analysis

We conducted a series of independent-sample *t* tests to evaluate differences in students’ perceived effectiveness, perceived realism, negative emotional response, and positive emotional response. This method was adequate to answer our research questions because *t* tests are considered a rigorous evaluation method for the comparison of means between two independent groups [97,98]. Independent sample *t* tests were particularly suitable to this study because they are considered the most general test to determine the significant differences between two independent samples [99]. Another reason for choosing independent-sample *t* tests for this study is that *t* tests have been widely used in campaign studies as the preferred analytical vehicle across health contexts [100,101,102,103,104]. Thus, we deployed SPSS (IBM Corp., Armonk, NY, U.A) to run the series of independent-sample *t* tests to answer our research question.

## 4. Results

A series of independent-sample *t* tests were conducted to answer the research question: How well do college students perceive theory-guided and tailored campaign interventions as effective, positive, and realistic? Overall, students responded positively to the interventions (see Table 2). Interestingly, gender differences were found in students’ evaluation of the campaigns. A significant gender difference was found on the perceived effectiveness of the “Working Out” ad (Ad No. 1) (*t*(424) = −2.21, *p* < 0.05), the “Paranoia” ad (Ad No. 4) (*t*(424) = −2.94, *p* < 0.01), the “Study Natural-Library” ad (Ad No. 5) (*t*(426) = −3.07, *p* < 0.01), and the “Study Natural-Outdoors” ad (Ad No. 6) (*t*(428) = −2.66, *p* < 0.01). A significant difference was also found in perceived realism by gender for the same four ads above: the “Working Out” ad (Ad No. 1) (*t*(430) = −2.06, *p* < 0.05), the “Paranoia” (Ad No. 4) ad (*t*(431) = −2.32, *p* < 0.05), the “Study Natural-Library” ad (Ad No. 5) (*t*(428) = −3.04, *p* < 0.01), and the “Study Natural-Outdoors” ad (*t*(429) = −2.54, *p* < 0.05). While no significant difference by gender was found regarding negative emotions, significant differences were found for positive emotions between male and female students’ evaluation of the “Study Natural-Library” ad (Ad No. 5) (*t*(430) = −2.41, *p* < 0.05).

## 5. Discussion

In this study, we aimed to investigate college students’ evaluation of theory-guided and tailored health campaign interventions in the NMUPS context. Specifically, we investigated whether college students would consider anti-NMUPS health campaigns developed under the theoretical guidance of the HBM and the TPB principles and tailored to their characteristics and preferences as effective and convincing. Our study is exploratory in nature. To the best of our knowledge, this is the first study examined the efficacy of theory-guided and tailored campaign interventions in changing college students’ attitudes and behavior towards NMUPS. Findings of our study can help address issues prevalent and persistent in the campaign literature: (1) a lack of theory-guided and tailored campaign interventions [30,31], (2) a lack of campaign studies that offer detailed information on how the campaign strategies and messages were designed and developed [84,86,87,88,89,105], and (3) a lack of campaign interventions in the context of NMUPS tailored to emerging adults such as college students [81,82,83].

The results of our study indicate that college students responded positively towards the campaign interventions. This suggests that theory-guided and tailored campaign strategies have great potential in addressing deep-rooted health issues such as engagement in NMUPS among college students. Future research could extend current understanding on effective intervention strategies by applying theory-guided and tailored strategies to guide intervention design and development and by further examining these methods in other health contexts using a larger and more diverse study population. It is important to note that while our finding indicates that the HBM and TPB frameworks are effective intervention strategies, we adopted these two theoretical frameworks based on suitability considerations. Future intervention studies could consider adopting or adapting theoretical underpinnings that are more reasonable to use in their specific research contexts.

The results also indicated that there was a gender difference in female and male college students’ evaluation of campaign interventions. Compared to their male counterparts, female college students were more likely to rate the “Working Out” (Ad No. 1), “Paranoia” (Ad No. 4), “Studying Natural-Library” (Ad No. 5), and the “Studying Natural-Outdoors” (Ad No. 6) ads as more effective and convincing. On the one hand, this finding resonates with current literature, which suggests that male college students are more likely to engage in NMUPS activities than their female counterparts [91,106]. On the other hand, by suggesting female and male students are not only different regarding their attitudes and behavior towards NMUPS, they are also different in how they respond to anti-NMUPS campaign interventions, our finding adds new insights into the research field. Overall, these insights can be adopted to guide future intervention studies.

Although the campaign strategies and messages in this study are theory-guided and tailored to college students, we did not adopt a gendered lens in designing and developing the interventions. It is important to acknowledge the fact that although college students’ attitudes and behavior towards NMUPS differ based on their gender, both female and male college students are contributors to and are influenced by campus social norms [45,58,107]. In other words, female college students who do not participate in NMUPS are peers to male students who engage in NMUPS. This suggests that students who are less likely to participate in NMUPS activities have the potential to influence the NMUPS-related attitudes and behavior of students who are more likely to partake in problematic NMUPS activities [45,58,107]. In other words, although male students might perceive anti-NMUPS campaigns as less effective than their female counterparts, their perceptions towards NMUPS might still be influenced by the attitudes and behavior of female college students who viewed the ads more positively [107].

To capitalize on our findings on gender differences regarding campaign evaluation, future studies could examine intervention strategies that are customized to male (or female) participants’ specific needs and preferences to see which intervention components are more effective in persuading this particular population to take positive health actions. As college students both contribute to and are influenced by campus social norms, future research could investigate how to best use positive social norms (e.g., participating in anti-NMUPS movements) to influence negative social norms that deteriorate college students’ academic performance and wellbeing. For instance, intervention studies could use campaign messages to accompany a tentative on-campus anti-NMUPS movement: “Using drugs to study will sour your brainpower and sabotage your manpower (or womanpower). Take a stand on making our campus drug-free: man up (or woman up), sign up, and show up!,” to investigate whether strategies such as this can further improve health campaigns’ abilities to change students’ NMUPS-related attitudes and behavior. Overall, while the findings of the current study fill critical voids in the literature, more research is needed to investigate effective campaign strategies that could further improve college students’ academic performance, health outcomes, and quality of life.

## 6. Limitations and Future Studies

While this study fills important voids in the literature, it is not without limitations. Overall, it is important to acknowledge that it is an exploratory study of theory-guided communication campaigns to prevent nonmedical use of prescription stimulants. First, though consistent with the participant pool’s gender ratio, the majority of our participants were females, which may limit the generalizability of our findings. Second, this study is cross-sectional in nature, which suggests that no causal relationships could be drawn from the findings. To address these limitations, future research could use a larger and more diverse student sample and adopt a longitudinal research design to further enrich the literature. Last but not least, although independent sample *t* tests helped us to address our research questions, we also need to acknowledge that the sample *t* tests did raise the concern about enlarging the possibility of type I errors. In our following study and any future studies, we shall consider ways of reducing type I errors, for instance, we could run regressions with dummy-coded variables when applicable.

## 7. Conclusions

This is the first study to explore the efficacy of adopting theory-guided and tailored campaign interventions in changing college students’ attitudes and behavior towards NMUPS. The findings of our study suggest that using theoretical frameworks to guide and tailor anti-NMUPS campaign design and development has the potential to induce positive changes in students. While this study fills important gaps in the literature, considering the progress needed to strengthen the research field, more research is needed to further identify effective strategies that could prevent college students’ participation in NMUPS as well as other detrimental health practices.

## Figures and Tables

**Table 1:** Demographic information of the respondents.

Measure	Items	Frequency	Percentage (%)
Gender	Male	108	25
	Female	328	74
Age	18–20	275	65
	21–23	149	33
	23 and above	10	2
Ethnicity	White	268	61
	Black	13	3
	Hispanic	71	16
	Asian	67	15
	Other	21	5
School Classification	Freshman	84	19
	Sophomore	126	29
	Junior	134	30
	Senior	94	21
	Other	3	1

**Table 2:** Results of *t* tests and descriptive statistics by gender.

Dependent Variables	Type of Ads	Male		Female		
		M	SD	M	SD	t
Perceived Effectiveness (1 = Definitely No; 4 = Definitely Yes)	Ad 1	2.28	0.57	2.42	0.53	−2.21 *
	Ad 2	2.23	0.66	2.35	0.61	−1.65
	Ad 3	2.45	0.80	2.49	0.77	−5.56
	Ad 4	2.21	0.71	2.44	0.71	−2.94 **
	Ad 5	2.16	0.80	2.41	0.69	−3.07 **
	Ad 6	2.14	0.81	2.35	0.68	−2.66 **
Perceived Realism (1 = Definitely No; 4 = Definitely Yes)	Ad 1	2.83	0.66	2.96	0.54	−2.06 *
	Ad 2	2.78	0.77	2.87	0.65	−1.20
	Ad 3	2.65	0.82	2.72	0.79	–0.84
	Ad 4	2.58	0.79	2.76	0.69	−2.32 *
	Ad 5	2.45	0.88	2.70	0.71	−3.04 **
	Ad 6	2.39	0.84	2.61	0.75	−2.54 *
Negative Emotion (1 = Not at All; 4 = Very much)	Ad 1	1.31	0.50	1.33	0.49	–0.35
	Ad 2	1.45	0.54	1.45	0.50	0.04
	Ad 3	1.45	0.61	1.37	0.55	1.33
	Ad 4	1.34	0.53	1.33	0.54	–0.03
	Ad 5	1.26	0.50	1.18	0.41	1.55
	Ad 6	1.20	0.42	1.19	0.39	0.35
Positive Emotion (1 = Not at All; 4 = Very much)	Ad 1	1.36	0.56	1.37	0.67	–0.10
	Ad 2	1.27	0.55	1.19	0.47	1.36
	Ad 3	1.23	0.50	1.26	0.57	–0.57
	Ad 4	1.15	0.38	1.14	0.41	0.10
	Ad 5	1.34	0.64	1.54	0.78	−2.41 *
	Ad 6	1.50	0.76	1.40	0.70	1.29

Ads: advertisements; M: mean; SD: standard deviation; *: *p* < 0.05, **: *p* < 0.01.

## References

[1] Munro B.A., Weyandt L.L., Marraccini M.E., Oster D.R. (2017). The relationship between nonmedical use of prescription stimulants, executive functioning and academic outcomes. Addict. Behav..

[2] McCabe S.E., West B.T., Teter C.J., Boyd C.J. (2014). Trends in medical use, diversion, and nonmedical use of prescription medications among college students from 2003 to 2013: Connecting the dots. Addict. Behav..

[3] Zullig K.J., Divin A.L. (2012). The association between non-medical prescription drug use, depressive symptoms, and suicidality among college students. Addict. Behav..

[4] Arria A.M., Caldeira K.M., Vincent K.B., O'Grady K.E., Cimini M.D., Geisner I.M., Fossos-Wong N., Kilmer J.R., Larimer M.E. (2017). Do college students improve their grades by using prescription stimulants nonmedically?. Addict. Behav..

[5] Verdi G., Weyandt L.L., Zavras B.M. (2014). Non-medical prescription stimulant use in graduate students. J. Atten. Disord..

[6] Jaconis M., Boyd S.J., Hartung C.M., McCrea S.M., Lefler E.K., Canu W.H. (2016). Sex differences in claimed and behavioral self-handicapping and ADHD symptomatology in emerging adults. Adhd Atten. Deficit. Hyperact. Disord..

[7] Wilens T.E., Carrellas N.W., Martelon M., Yule A.M., Fried R., Anselmo R., McCabe S.E. (2017). Neuropsychological functioning in college students who misuse prescription stimulants. Am. J. Addict..

[8] White C., Kolble R., Carlson R., Lipson N. (2005). The impact of a health campaign on hand hygiene and upper respiratory illness among college students living in residence halls. J. Am. Coll. Health.

[9] Cruz S., Sumstine S., Mendez J., Bavarian N. (2017). Health-compromising practices of undergraduate college students: Examining racial/ethnic and gender differences in characteristics of prescription stimulant misuse. Addict. Behav..

[10] Zander M.E., Norton-Baker M., De Young K.P., Looby A. (2016). The role of anonymity in determining the self-reported use of cocaine and nonmedical prescription stimulant use among college students. Subst. Use Misuse.

[11] Rabiner D.L., Anastopoulos A.D., Costello E.J., Hoyle R.H., McCabe S.E., Swartzwelder H.S. (2008). Motives and perceived consequences of nonmedical ADHD medication use by college students. J. Atten. Disord..

[12] Drug Prevention. http://www.valdosta.edu/about/news/releases/2010/10/volunteerweek-101910.php.

[13] Snyder L.B., Hamilton M.A., Mitchell E.W., Kiwanuka-Tondo J., Fleming-Milici F., Proctor D. (2004). A meta-analysis of the effect of mediated health communication campaigns on behavior change in the United States. J. Health Commun..

[14] Anker A.E., Feeley T.H., McCracken B., Lagoe C.A. (2016). Measuring the effectiveness of mass-mediated health campaigns through meta-analysis. J. Health Commun..

[15] Friedman A.L., Kachur R.E., Noar S.M., McFarlane M. (2016). Health communication and social marketing campaigns for sexually transmitted disease prevention and control: What is the evidence of their effectiveness?. Sex. Transm. Dis..

[16] Babalola S., Figueroa M.E., Krenn S. (2017). Association of mass media communication with contraceptive use in sub-Saharan Africa: A meta-analysis of demographic and health surveys. J. Health Commun..

[17] Lombardo A.P., Léger Y.A. (2007). Thinking about “Think Again” in Canada: Assessing a social marketing HIV/AIDS prevention campaign. J. Health Commun..

[18] Thackeray R., Neiger B.L. (2000). Establishing a relationship between behavior change theory and social marketing: Implications for health education. J. Health Educ..

[19] Weinreich N.K. (1999). Hands-on Social Marketing: A Step-by-Step Guide.

[20] Glanz K., Rimer B.K. (2005). Theory at a Glance. A Guide for Health Promotion Practice.

[21] Hastings G. (2007). Social Marketing: Why Should the Devil Have All the Best Tunes?.

[22] Guyatt G.H., Oxman A.D., Kunz R., Vist G.E., Falck-Ytter Y., Schünemann H.J. (2008). What is “quality of evidence” and why is it important to clinicians?. BMJ.

[23] Sena E.S., Currie G.L., McCann S.K., Macleod M.R., Howells D.W. (2014). Systematic reviews and meta-analysis of preclinical studies: Why perform them and how to appraise them critically. J. Cereb. Blood Flow Metab..

[24] White H., Waddington H. (2012). Why do we care about evidence synthesis? An introduction to the special issue on systematic reviews. J. Dev. Eff..

[25] Dann G., Nash D., Pearce P. (1988). Methodology in tourism research. Ann. Tour. Res..

[26] Record R.A., Harrington N.G., Helme D.W., Savage M.W. (2018). Using the Theory of Planned Behavior to guide focus group development of messages aimed at increasing compliance with a tobacco-free policy. Am. J. Health Promot..

[27] Cameron D., Epton T., Norman P., Sheeran P., Harris P.R., Webb T.L., Julious S.A., Brennan A., Thomas C., Petroczi A. (2015). A theory-based online health behaviour intervention for new university students (U@Uni:LifeGuide): Results from a repeat randomized controlled trial. Trials.

[28] Ortiz R.R., Shafer A. (2018). Unblurring the lines of sexual consent with a college student-driven sexual consent education campaign. J. Am. Coll. Health.

[29] Gray J.B. (2011). Theory guiding communication campaign praxis: A qualitative elicitation study comparing exercise beliefs of overweight and healthy weight college students. Qual. Res. Rep. Commun..

[30] Truong D., Vu D., Nam H., Kubacki K., Rundle-Thiele S. (2017). Reviewing research evidence for social marketing: Systematic literature reviews. Formative Research in Social Marketing: Innovative Methods to Gain Consumer.

[31] Truong V.D. (2014). Social Marketing: A Systematic Review of Research 1998–2012. Soc. Mark. Q..

[32] Luca N.R., Suggs L.S. (2013). Theory and model use in social marketing health interventions. J. Health Commun..

[33] Noar S.M., Harrington N.G., Aldrich R.S. (2009). The role of message tailoring in the development of persuasive health communication messages. Ann. Int. Commun. Assoc..

[34] Wakefield M.A., Loken B., Hornik R.C. (2010). Use of mass media campaigns to change health behaviour. Lancet.

[35] Latimer A.E., Brawley L.R., Bassett R.L. (2010). A systematic review of three approaches for constructing physical activity messages: What messages work and what improvements are needed?. Int. J. Behav. Nutr. Phys. Act..

[36] Arria A.M., Geisner I.M., Cimini M.D., Kilmer J.R., Caldeira K.M., Barrall A.L., Vincent K.B., Fossos-Wong N., Yeh J.-C., Rhew I. (2018). Perceived academic benefit is associated with nonmedical prescription stimulant use among college students. Addict. Behav..

[37] Kinman B.A., Armstrong K.J., Hood K.B. (2017). Perceptions of risks and benefits among nonprescription stimulant consumers, diverters, and non-users. Subst. Use Misuse.

[38] Smith T.E., Martel M.M., DeSantis A.D. (2017). Subjective report of side effects of prescribed and nonprescribed psychostimulant use in young adults. Subst. Use Misuse.

[39] Liang H., Xue Y., Berger B.A. (2006). Web-based intervention support system for health promotion. Decis. Support Syst..

[40] Vrecko S. (2013). Just how cognitive is “cognitive enhancement”? on the significance of emotions in university students' experiences with study drugs. Ajob Neurosci..

[41] Schelle K.J., Olthof B.M.J., Reintjes W., Bundt C., Gusman-Vermeer J., van Mil A.C.C.M. (2015). A survey of substance use for cognitive enhancement by university students in the Netherlands. Front. Syst. Neurosci..

[42] Teter C.J., McCabe S.E., LaGrange K., Cranford J.A., Boyd C.J. (2006). Illicit use of specific prescription stimulants among college students: Prevalence, motives, and routes of administration. Pharmacotherapy.

[43] Desantis A.D., Hane A.C. (2010). “Adderall is definitely not a drug”: Justifications for the illegal use of ADHD stimulants. Subst. Use Misuse.

[44] Bennett T., Holloway K. (2017). Motives for illicit prescription drug use among university students: A systematic review and meta-analysis. Int. J. Drug Policy.

[45] Bavarian N., McMullen J., Flay B.R., Kodama C., Martin M., Saltz R.F. (2017). A mixed-methods approach examining illicit prescription stimulant use: Findings from a Northern California University. J. Prim. Prev..

[46] Ram S., Hussainy S., Henning M., Stewart K., Jensen M., Russell B. (2017). Attitudes toward cognitive enhancer use among New Zealand tertiary students. Subst. Use Misuse.

[47] LaBelle S. (2018). College Students' intent to intervene when a peer is engaging in nonmedical use of prescription stimulants: An application of the Theory of Planned Behavior. Subst. Use Misuse.

[48] Thompson K., Burgess J., MacNevin P.D. (2018). An evaluation of e-CHECKUP TO GO in Canada: The mediating role of changes in social norm misperceptions. Subst. Use Misuse.

[49] Polonec L.D., Major A.M., Atwood L.E. (2006). Evaluating the believability and effectiveness of the social norms message "Most Students Drink 0 to 4 Drinks When They Party". Health Commun..

[50] Blevins C.E., Stephens R., Abrantes A.M. (2017). Motives for prescription stimulant misuse in a college sample: Characteristics of users, perception of risk, and consequences of use. Subst. Use Misuse.

[51] McNiel A.D., Muzzin K.B., DeWald J.P., McCann A.L., Schneiderman E.D., Scofield J., Campbell P.R. (2011). The nonmedical use of prescription stimulants among dental and dental hygiene students. J. Dent. Educ..

[52] Zhang X., Cowling D.W., Tang H. (2010). The impact of social norm change strategies on smokers' quitting behaviours. Tob. Control.

[53] Desantis A., Noar S.M., Webb E.M. (2010). Speeding through the Frat House: A qualitative exploration of nonmedical ADHD stimulant use in fraternities. J. Drug Educ..

[54] Schultz N.R., Silvestri M.M., Correia C.J. (2017). Diversion of prescription stimulants among college students: An initial investigation of injunctive norms. Addict. Behav..

[55] Bavarian N., Flay B.R., Ketcham P.L., Smit E. (2013). Illicit use of prescription stimulants in a college student sample: A theory-guided analysis. Drug Alcohol Depend..

[56] Hanson C.L., Burton S.H., Giraud-Carrier C., West J.H., Barnes M.D., Hansen B. (2013). Tweaking and tweeting: Exploring Twitter for nonmedical use of a psychostimulant drug (Adderall) among college students. J. Med. Internet Res..

[57] Fogel J., Shlivko A. (2016). Reality television programs are associated with illegal drug use and prescription drug misuse among college students. Subst. Use Misuse.

[58] Bavarian N., Flay B.R., Ketcham P.L., Smit E. (2015). The illicit use of prescription stimulants on college campuses. Health Educ. Behav..

[59] Chen L.-Y., Crum R.M., Strain E.C., Alexander G.C., Kaufmann C., Mojtabai R. (2016). Prescriptions, nonmedical use, and emergency department visits involving prescription stimulants. J. Clin. Psychiatry.

[60] McCabe S.E., Knight J.R., Teter C.J., Wechsler H. (2005). Non-medical use of prescription stimulants among US college students: Prevalence and correlates from a national survey. Addiction.

[61] DeSantis A. (2007). Inside Greek U.: Fraternities, Sororities, and the Pursuit of Pleasure, Power, and Prestige.

[62] Herman-Stahl M.A., Krebs C.P., Kroutil L.A., Heller D.C. (2007). Risk and protective factors for methamphetamine use and nonmedical use of prescription stimulants among young adults aged 18 to 25. Addict. Behav..

[63] McCabe S.E., Teter C.J., Boyd C.J. (2006). Medical use, illicit use, and diversion of abusable prescription drugs. J. Am. Coll. Health.

[64] Silvestri M.M., Correia C.J. (2016). Normative influences on the nonmedical use of prescription stimulants among college students. Psychol. Addict. Behav..

[65] McCabe S.E., Teter C.J., Boyd C.J. (2006). Medical use, illicit use and diversion of prescription stimulant medication. J. Psychoact. Drugs.

[66] Fischer B., Bibby M., Bouchard M. (2010). The global diversion of pharmaceutical drugs non-medical use and diversion of psychotropic prescription drugs in North America: A review of sourcing routes and control measures. Addiction.

[67] Viswanath K., Orleans C.T., Glanz K., Rimer B.K. (2008). Health Behavior and Health Education: Theory, Research, and Practice.

[68] Janz N.K., Becker M.H. (1984). The Health Belief Model: A decade later. Health Educ. Q..

[69] Rosenstock I.M., Glanz K., Lewis F., Rimer B. (1990). The Health Belief Model: Explaining health behaviour through Expectancies. Health Behaviour and Health Education: Theory, Research, and Practice.

[70] Rosenstock I.M. (1974). Historical origins of the Health Belief Model. Health Educ. Monogr..

[71] Fishbein M., Yzer M.C. (2003). Using theory to design effective health behavior interventions. Commun. Theory.

[72] Noar S.M. (2006). A 10-Year retrospective of research in health mass media campaigns: Where do we go from here?. J. Health Commun..

[73] Noar S.M., Benac C.N., Harris M.S. (2007). Does tailoring matter? Meta-analytic review of tailored print health behavior change interventions. Psychol. Bull..

[74] Shafer A., Cates J.R., Diehl S.J., Hartmann M. (2011). Asking Mom: Formative research for an HPV vaccine campaign targeting mothers of adolescent girls. J. Health Commun..

[75] Eisen M., Zellman G.L., McAlister A.L. (1992). A health belief model-social learning theory approach to adolescents' fertility control: Findings from a controlled field trial. Health Educ. Q..

[76] Champion V.L., Celette S.S., Glanz K., Rimer B.K., Viswanath K. (2008). The health belief model. Health Behavior and Health Education: Theory, Research, and Practice.

[77] Rosenstock I.M. (1966). Why People Use Health Services. Milbank Q..

[78] Ajzen I. (2002). Perceived behavioral control, self-efficacy, locus of control, and the theory of planned behavior. J. Appl. Soc. Psychol..

[79] Ajzen I. (1991). The theory of planned behavior. Organ. Behav. Hum. Decis. Process..

[80] Gallucci A., Martin R., Beaujean A., Usdan S. (2015). An examination of the misuse of prescription stimulants among college students using the theory of planned behavior. Psychol. Health Med..

[81] Berg C.J., Lessard L., Parelkar P.P., Thrasher J., Kegler M.C., Escoffery C., Goldade K., Ahluwalia J.S. (2011). College student reactions to smoking bans in public, on campus and at home. Health Educ. Res..

[82] Reisinger K.B., Rutledge P.C., Conklin S.M. (2016). Study drugs and academic integrity: The role of beliefs about an academic honor code in the prediction of nonmedical prescription drug use for academic enhancement. J. Coll. Stud. Dev..

[83] Kwan M.Y., Arbour-Nicitopoulos K.P., Lowe D., Taman S., Faulkner G.E. (2010). Student reception, sources, and believability of health-related information. J. Am. Coll. Health.

[84] Stead M., Angus K., Langley T., Katikireddi S., Hinds K., Hilton S., Lewis S., Thomas J., Campbell M., Young B. (2019). Mass media to communicate public health messages in six health topic areas: A systematic review and other reviews of the evidence. Public Health Res..

[85] Robinson M.N., Tansil K.A., Elder R.W., Soler R.E., Labre M.P., Mercer S.L., Eroglu D., Baur C., Lyon-Daniel K., Fridinger F. (2014). Mass media health communication campaigns combined with health-related product distribution: A community guide systematic review. Am. J. Prev. Med..

[86] Torloni M.R., Brizuela V., Betran A.P. (2020). Mass media campaigns to reduce unnecessary caesarean sections: A systematic review. BMJ Glob. Health.

[87] Suman A., Armijo-Olivo S., Deshpande S., Marietta-Vasquez J., Dennett L., Miciak M., Reneman M., Werner E.L., Straube S., Buchbinder R. (2020). A systematic review of the effectiveness of mass media campaigns for the management of low back pain. Disabil. Rehabil..

[88] Englund T.R., Zhou M., Hedrick V.E., Kraak V.I. (2020). How branded marketing and media campaigns can support a healthy diet and food well-being for Americans: Evidence for 13 campaigns in the United States. J. Nutr. Educ. Behav..

[89] Chan L., O'Hara B., Phongsavan P., Bauman A., Freeman B. (2020). From ‘likes’ to quit attempts: A review of evaluation metrics used in digital and traditional tobacco control campaigns. J. Med. Internet Res..

[90] Bartlett J.E., Kotrlik J.W., Higgins C.C. (2001). Organizational research: Determining appropriate sample size in survey research appropriate sample size in survey research. Inf. Technol. Learn. Perform. J..

[91] McCabe S.E., Morales M., Cranford J.A., Delva J., McPherson M.D., Boyd C.J. (2007). Race/ethnicity and gender differences in drug use and abuse among college students. J. Ethn. Subst. Abus..

[92] Smith T.E., DeSantis A.D., Martel M.M. (2017). Gender Differences in Nonprescribed Psychostimulant Use in Young Adults. Subst. Use Misuse.

[93] Becker M.H. (1974). The health belief model and personal health behavior. Health Commun. Monogr..

[94] Hovland C., Weiss W. (1951). The influence of source credibility on communication effectiveness. Public Opin. Q..

[95] Case K.R., Lazard A.J., Mackert M.S., Perry C.L. (2018). Source credibility and e-cigarette attitudes: Implications for tobacco communication. Health Commun..

[96] Fishbein M., Hall-Jamieson K., Zimmer E., von Haeften I., Nabi R. (2002). Avoiding the boomerang: Testing the relative effectiveness of antidrug public service announcements before a national campaign. Am. J. Public Health.

[97] Fishbein M., Cappella J., Hornik R., Sayeed S., Yzer M., Ahern R.K., Crano W.D., Burgoon M. (2002). The role of theory in developing effective antidrug public service announcements. Mass Media and Drug Prevention: Classic and Contemporary Theories and Research.

[98] Archie J.W. (1985). Statistical analysis of heterozygosity data: Independent sample comparisons. Evolution.

[99] Pandey R. (2015). Commonly used t-tests in medical research. J. Pract. Cardiovasc. Sci..

[100] Esmaeilzadeh S., Ashrafi-rizi H., Shahrzadi L., Mostafavi F. (2018). A survey on adolescent health information seeking behavior related to high-risk behaviors in a selected educational district in Isfahan. PLoS ONE.

[101] Leyden K.M., Reger-Nash B., Bauman A., Bias T. (2008). Changing the hearts and minds of policy makers: An exploratory study associated with the West Virginia Walks Campaign. Am. J. Health Promot..

[102] Livingston J.D., Tugwell A., Korf-Uzan K., Cianfrone M., Coniglio C. (2013). Evaluation of a campaign to improve awareness and attitudes of young people towards mental health issues. Soc. Psychiatry Psychiatr. Epidemiol..

[103] Sundstrom B., Billings D., Smith E., Ferrara M., Albert B., Suellentrop K. (2019). Evaluating the Whoops Proof S.C. campaign: A pair-matched group pretest–posttest quasi-experimental study. Matern. Child Health J..

[104] Kite J., Grunseit A., Li V., Vineburg J., Berton N., Bauman A., Freeman B. (2019). Generating engagement on the Make Healthy Normal Campaign Facebook page: Analysis of Facebook analytics. JMIR Public Health Surveill..

[105] Mealy R.N., Richardson L.A., Miller B., Smith M., Juvancic-Heltzel J.A. (2019). Exercise is Medicine^®^: Knowledge and awareness among exercise science and medical school students. Int. J. Exerc. Sci..

[106] McCabe S.E., Teter C.J., Boyd C.J. (2004). The use, misuse and diversion of prescription stimulants among middle and high school students. Subst. Use Misuse.

[107] Benson K., Flory K., Humphreys K.L., Lee S.S. (2015). Misuse of stimulant medication among college students: A comprehensive review and meta-analysis. Clin. Child Fam. Psychol. Rev..

